# The Drinker’s Effect on the Social Environment: A Conceptual Framework for Studying Alcohol’s Harm to Others

**DOI:** 10.3390/ijerph7041855

**Published:** 2010-04-21

**Authors:** Robin Room, Jason Ferris, Anne-Marie Laslett, Michael Livingston, Janette Mugavin, Claire Wilkinson

**Affiliations:** 1 Turning Point Alcohol and Drug Centre, 54–62 Gertrude St., Fitzroy, Victoria 3065, Australia; E-Mails: jasonf@turningpoint.org.au (J.F.); annel@turningpoint.org.au (A.L.); michaell@turningpoint.org.au (M.L.); janettem@turningpoint.org.au (J.M.); clairew@turningpoint.org.au (C.W.); 2 School of Population Health, University of Melbourne, Parkville, Victoria 3010, Australia

**Keywords:** alcohol, harm to others, interpersonal problems, effects of drinking

## Abstract

The paper considers conceptual and methodological issues in studying the scope of alcohol’s harm to others. Reasons are suggested for the relative neglect of the topic. The approaches in two relevant research traditions are considered: population surveys on alcohol problems, and economic cost of alcohol studies. Ways of conceptualizing and measuring aspects of the drinker’s effects on others are considered, in terms of main types of relationship with the other, and in terms of major societal response institutions. The main types of data tend to measure different levels of severity, with population survey data dominated by less severe problems, and response institution data by more severe problems; so both are needed for a three-dimensional view. Research questions for the field and its policy significance are noted.

## 1. Introduction

Drinking often results in harm not only to the drinker, but also to others around the drinker. A traffic crash caused by a drinking driver may kill passengers, the other driver or pedestrians. An elderly couple walking home at night may be threatened by carousing teenagers. A child may be left stranded when an adult supposed to pick the child up from preschool instead overstays an after-work drinking session. A sober bystander attempting to separate two drunks fighting may himself be injured when they turn on him. An adult daughter may find herself at her wit’s end over the drinking of her live-in father. A small firm may be driven into bankruptcy by mistakes or misdeeds of its employees when they have been drinking on the job. A pregnant woman may continue her heavy drinking and harm her baby. All of these instances are examples of ways in which someone’s drinking may adversely affect someone else.

As will be discussed, the literature on the scope of the harm from drinking to the social environment of the drinker is small, though there are substantial literatures on some specific areas of harm, such as from drinking driving, and from violence, including domestic violence. This paper discusses conceptual and methodological issues in studying the scope of alcohol’s harm to others.

## 2. Behind the Neglect of Alcohol’s Harm to Others

In the modern literature on alcohol problems the harms which are best measured are the effects of drinking on health. Estimates of the role of alcohol in each of a variety of diseases now draw on meta-analyses based in turn on dozens of prospective and case-control studies [1]. The dominance in data and research of health studies reflects higher investments in many countries in health research than in research in such other areas as welfare or criminology. As well as a rich tradition of general-population health surveys, the studies draw on substantial investments in general medical record-keeping.

But health records usually focus on characteristics of an individual defined as the patient. The International Classification of Diseases is a catalogue built around the body of the individual patient. Even for injuries, where the environment is coded, the codes pay attention primarily to the inanimate context of the injury: the injury was from an automobile, a particular poison, a fall or a fire. The classification, and the health records in which such classifications are used, are largely blind to the condition of other humans as an element of the context of the disorder.

The emphasis on harms to the drinker also arises from the general bias towards methodological individualism, with samples designed in terms of isolated individuals, in research methods and questions. Population surveys typically interview only one person per household, and sampled in such a way as to limit any cross-contamination of responses between respondents. Greater attention is often paid to the life history of the individual than to factors in the individual’s social environment. Measuring alcohol’s harm to others requires at a minimum measuring drinking and associated behaviours of one person and harm to another.

There are some realms of harm to others where there is more attention to social interactions and human elements of the environment. Police reports on homicides or sexual assaults typically pay detailed attention to potential perpetrators, and in general to the human context of the crime. Child welfare investigations parse all relationships and every member in the child’s family. But the presence of drinking or intoxication is often only fitfully recorded in the case notes.

This general situation differs substantially from a century ago. A marital partner’s inebriety was an accepted ground for divorce, which meant that official statistics were available. “Worker’s compensation systems collected data on findings that the claimant had been drinking…. Life insurance companies routinely collected data on an applicant’s ‘drinking habits’” [2]. In general, the role of alcohol in social problems was more routinely recorded.

What happened in the interim were two big social changes. By the mid-20th century, there had been a strong generational reaction against the temperance movement. Alcohol’s involvement in social problems—the adverse effects of the drunkard’s drinking upon the family, the economic loss to business from employee drinking, the idea that it was alcohol which filled the prisons—had been a staple of temperance movement campaigns and rhetoric [3], and in the reaction against temperance there was a tendency to deny or turn away from this. For more than a generation, textbooks on criminology, using a very stringent definition of causation, maintained that drinking was not a causal factor in violence or crime [4]. The idea that alcohol caused harm to others came to be seen as an old-fashioned temperance idea. Even in the medical literature on alcohol’s harm to the drinker, there was a wave of denial of relationships by respectable scholars, for instance of alcohol and liver cirrhosis [5], that now seems incredible.

The other change was in ideas and boundaries of privacy. Among the several social trends which contributed to the move to “no-fault divorce” in the 1960s and after, and thus to the loss of statistics about alcohol’s contribution to divorce, one change was an expansion in the realm of privacy, in 1965 elevated in the USA to the state of a constitutional right [6]. During the temperance period being a drinker or not had been, to a considerable extent, a matter of public status; the change in the boundary of the private meant that whether and what one drank became a private matter; one’s drinking habits, so long as they were not flaunted in public, moved into the realm of the personal, something which was not anyone else’s business. If a family member was distressed by another family member’s drinking, it should be discussed with fellow-sufferers behind closed doors in Al-Anon, not made a public issue. “Drinking-related problems were redefined more as private than as public matters [7], and drinking itself came to be seen as in the sphere of private life rather than as a matter of public interest” [2].

These social changes have had a long persistence, even after the tide has clearly changed on recognizing the serious effects of drinking on the drinker him- or herself. One early breach in the neglect of alcohol’s effect on others was in the area of drinking driving. By the 1960s, the serious effects of drinking driving on others were recognized and became arguments for policy change [7]. In other areas, the recognition of harm to others from drinking has been much weaker, and the societal response much more halting. The feminist concern to counter intimate violence prevailed over norms of the privacy of home life. Against the preference of some strands of feminist thought, which have feared that attention to the male assailant’s drinking will offer him an excuse, this has eventually led to some focus on the role of drinking, so that police in Victoria, Australia, for instance, record alcohol’s involvement more reliably for domestic violence than for street violence. But despite increasing public concern about “binge drinking” and street violence, direct police data on the contribution of drinking to violence in public places is still scanty.

## 3. Conceptualizing Alcohol’s Harm to Others

The harms from drinking are conventionally discussed in terms of health problems and social problems [8]. In fact, there is considerable overlap in the two domains, particularly concerning injuries. A loose equation is sometimes made between health problems as problems for the drinker and social problems as problems for others besides the drinker. But this equation is flawed. Some alcohol-related health problems occur to others than the drinker. This is the case for injuries, for foetal alcohol effects, and for mental disorders to family members resulting from the drinker’s behaviour. On the other side, a social problem may be a problem for the drinker, whether or not there is a problem for someone else: defaults in one’s work because of drinking may result in the drinker being fired, whether or not there is a loss of productivity for the workplace.

However, most social problems with drinking involve some harm, perceived or tangible, to another person. Someone other than the drinker is perceived or perceives him/herself to be adversely affected by the drinking, and a social problem with drinking often involves some response by the other person which in turn adversely affects the drinker. Most social problems with drinking are thus inherently interactional.

It should be noted that in most circumstances human perception and definition are thus inherently involved in whether person A’s drinking is defined as harmful to person B. A may not perceive there to be a problem for B. A may not perceive his or her drinking to have any relation to it. Or A may not know that B considers A’s drinking to have created problems for B. Naturally, B may have his or her own permutations on these possibilities. An observer may have a third set of perceptions and definitions. Thus, an old USA study of 252 domestic physical assaults where the police were called found that the putative assaulter was alleged by the complainant to have been drinking in 40% of the cases, but that the police judged that the person had been drinking in only 21% [9].

The evidence which is commonly available on alcohol’s harm to others draws from a variety of perspectives on the occurrence of a problem and drinking’s relationship to it. Data from police or hospital records are formally recorded by professionals or the relevant organization’s clerical staff. They may represent professional observation. But they may also or instead reflect what is volunteered or answered by one or another party to a situation. Data from surveys reflect answers by a drinker or by someone adversely affected by a drinker, but built into the questions or answers may be the respondent’s report of what someone else said.

A book edited by Klingemann and Gmel [10] is the most sustained discussion yet of the nature of and data on social consequences of alcohol consumption. Chapters of the book on substantive domains cover harm to relatives; “friends and the close social environment”; the impact on work and education; public order and safety; and accidents, suicide and violence. Underneath these rubrics we can discern an organization partly in terms of major social roles (work, family, friendship), and partly in terms of major social institutions (welfare and child protection; the workplace; police; and accident and emergency services).

As we consider the traditions of research on social harms from drinking, versions of these two underlying dimensions of organisation—by major social roles and by major societal response institutions—are a recurrent theme. One reason the major social institutions figure heavily in the conceptual organization is that they are the main avenues of societal response to social problems, and thus generate the records on which most research on serious consequences is based. The major social roles look at social consequences instead from the perspective of the individual involved—usually the drinking individual: how has the drinking impinged on major areas of his or her life? The obverse of these social roles is sets of persons in interaction with the drinker: employer and workmates; spouse/partner, household members, relatives; friends, neighbours and acquaintances. What must be added to these sets is the indefinitely large set of strangers who may be affected by the drinker: those whose sleep is disturbed by the drunkard’s noisy path home, passersby caught up in a drunken fight, owners of “lemon” automobiles supposed to be more commonly built when the weekend drinkers were missing from the assembly line. The adverse effect may come from collective acts or threats, or may come from an individual drinker. Thinking in terms of the individual drinker, many of these constituencies are affected by the drinker’s comportment as an actor in public spaces [11], carrying out what we may describe as a role as public person: someone expected to act with decorum and civil inattention, not disturbing the peace or intruding on others’ space.

**Figure 1.**
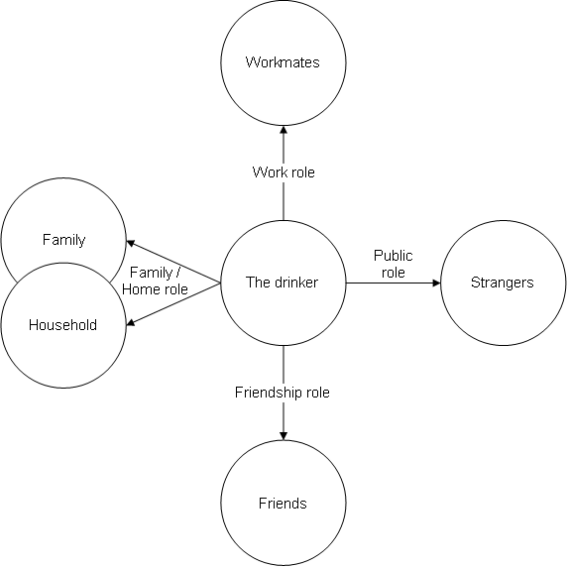
The drinker’s impact on others—main types of relationships.

Figure 1 summarizes this view of the problems for others from a person’s drinking in terms of the interactions between the drinker and five principal constituencies, involving four main sets of roles. The left and bottom relationships might be thought of in terms of the sphere of private life, and the top and right relationships as in the sphere of public life. Implicitly, the focus in Figure 1 is on problems at the level of the individual or the social interaction or relationship. Harm to others from drinking can also occur at the level of a larger collectivity, including a whole society. This phenomenon is easier to see in extreme cases in small societies; a number of examples can be found of serious consequences for the collectivity from prevalent heavy drinking by some members of the society. Thus, concerning the effects of free access to alcohol on Aboriginal society in Western Australia, Sackett notes not only the rise in injuries, health problems and child neglect, but also the adverse effects at a collective level on religious practices, social rules and ritual activities [12,13, pp. 152–154]. Conceptually, the tradition of economic studies of the social cost of alcohol includes harm to the society as a whole, for instance in calculating as an “indirect cost” the lost productivity to the society from those who have died early due to alcohol-related illness. Although the main focus in the present discussion is on the individual and interactional levels—harms to particular individuals or small groups or settings from particular drinkers—we also consider how a focus on harm to specific others relates to the concepts and measures in traditions of social cost studies.

## 4. Relevant Comprehensive Approaches to Measuring Harms from Drinking

There are two main alcohol-specific literatures in which there have been attempts to be comprehensive about alcohol-related harms: the survey research tradition of asking about alcohol problems, and the tradition of “cost-of-illness” studies of the social costs of alcohol. The two literatures look at alcohol-related harms through very different windows.

### 4.1. Survey Research Traditions

The primary strand in the survey research tradition has asked the drinker about problems from his or her own drinking. These have included a wide range of problems, personal and social. As noted above, social problems from drinking tend to be inherently interactional, so that when a drinker is answering that his drinking has harmed his marriage or home life, for instance, it is very likely there is another who considers him/herself to have been adversely affected by the drinking.

A typical listing of problem areas relevant here would include problems related to the respondent’s drinking with a spouse/partner, with relatives, with friends or neighbours, on the job or with workmates, and with the police [14,15]. Sometimes the first three of these categories have been combined in an “Interpersonal Consequences” score. Two further problem areas in this tradition, Problems with Finances and Belligerence (getting in arguments or fights, *etc.*), are also somewhat relevant.

Implicit in this survey research tradition is a conceptualization of social problems from drinking in terms of default in major social roles—the roles specified in Figure 1. The perspective is, of course, the limited perspective of the drinker. Many of the survey items ask about the drinker’s perception of others’ reactions, often without being tied to specific events or circumstances. Thus, even though it gets scored as a “mild problem” with the spouse, we do not know what lies behind a positive response to the survey item, “My wife indicated I should cut down on my drinking” [15]. While the tradition of asking drinkers about their social problems with drinking is clearly relevant to drinking’s harms to others, it is thus not a direct and precise way of measuring alcohol’s harm to others.

Overlapping this tradition has been a line of analysis of patterns of informal social control of drinking—for instance, suggestions to the drinker to cut down drinking [16,17]. The potential harm to the other from the drinking is usually not explicit in these studies, although the drinker’s problems to which the other is reacting can clearly be substantial [18,19]. Studies in this tradition have paid some attention to the patterning of suggestions and pressure between genders, and across generations within the family.

There are also growing traditions of survey studies which focus more on specific kinds of interpersonal problems – for instance, the literature on alcohol in partner violence [20]. In studies in this area, specific data on adverse effects on a partner related to the other’s drinking can be gathered from the victim as well as from the aggressor, although not usually in the same couple.

A secondary strand in the survey research tradition has looked at alcohol-related harms specifically from the perspective of the other—whether in the role of victim or (less often) of bystander. In this strand, while the relation of the drinker to the respondent is often not clear, the focus is not on fuzzily-defined “drinking problems”, but instead squarely on harm experienced by the respondent from others’ drinking. “The focus is usually explicitly on concrete events, whereas items in own-drinking problem series often concern conditions, or are commonly interpreted as indicators of a condition” [15]. The paper in this issue by Greenfield *et al.* [21] is a current example of such an analysis.

Analyses of the questions have often stayed at the item level [22,23], without consideration of problem domains. In the earliest modern study in this tradition, Fillmore [24] did make an ad-hoc division into subscales: obnoxious behaviour, property damage, family and friend problems, violence, accidents, and employment threatened. The “obnoxious” subscale combines items about observation of public disorder and about a party being spoiled, regardless of whether there was an impact on the respondent, with items about the effect on the respondent’s home life. Both the Mäkelä *et al.* [23] and the Eliany *et al.* [22] series confined themselves to harm directly to the respondent. Conceptually, analyses in this tradition have not distinguished clearly between the private and the public spheres.

In existing studies, the questions about harm from others’ drinking have been a relatively minor part of a broad-ranging questionnaire. The range of topics to be covered has tended to mean only a shallow coverage of each one, particularly in the era of the 20-minute telephone interview. The questions typically pick up adverse effects of drinking in both the public and the private spheres.

This conceptual paper is written in the context of a study which includes a population survey specifically devoted to measuring alcohol’s harm to others [25]. In the survey, we are asking about the harms to a single person, the respondent, from a range of drinkers. To increase the specificity and validity of the responses, minimising recall bias, we confined the time period to the previous twelve months. Different sections of the questionnaire cover drinkers in different forms of relationship with the respondent. The domains of relationship covered in the study are those of Figure 1. But the survey’s approach is from the perspective of the other, rather than the drinker. The respondent is asked about adverse effects on him or her from drinkers in each of the role relationships shown in Figure 1.

### 4.2. The Cost of Alcohol Tradition

The other main relevant broad-ranging tradition of work derives from the “cost of illness” tradition in economics. While, as its name implies, this tradition originated specifically with respect to disease and the health system [26], from the first it took a broad view of the range of social costs involved, and thus it has been fairly readily adaptable to studies of the social costs of alcohol and other drugs— where much of the harm is not attributable to a disease [27]. The basic building-blocks of cost of alcohol studies are derived from register data, that is, the records of case-by-case operation of the major institutions of societal response to problems—hospitals and the health system, the police and criminal court system, the unemployment and welfare systems, and so on. To these building blocks are applied estimates of the alcohol-attributable fraction of the caseload, either directly derived from alcohol codes in the system’s records, or estimated on the basis of a variety of other data.

In principle, the focus is particularly on the costs to others than the drinker, defined in economic jargon as “negative externalities”: “Negative externalities occur when individuals or firms undertake actions which impose costs on other individuals or firms, while providing no, or insufficient, compensation to those who bear these extra costs.” [28, p. 8] However, in many cost of alcohol analyses some costs to the drinker are also included, with the argument that the drinker could or did not fully take into account these potential costs in his or her choices about drinking.

A small tradition within economics, critical of the general cost-of-illness tradition discussed next, has endeavoured to confine the estimated costs to strictly-defined “external costs”, that is, costs imposed on others by the alcohol consumption of the drinker [29,30]. However, these studies have been quite rigid in excluding costs which they do not regard as true externalities. Thus Heien & Pittman [29] exclude costs to others in the drinker’s family on the grounds that these “are basically internalized within the family”. In their rigid approach to what constitutes an “externality”, Heien and Pittman also exclude injuries to a passenger in a drink driver’s car on the grounds the passenger “has accepted the risk of riding with an abuser”. Costs of crime are excluded because, in their view, “the question of a causal relation between crime and alcohol abuse is tenuous at best”. After such exclusions, the main cost left in Heien and Pittman’s accounting is costs of fatalities and injuries of those in a drinking-driving crash who are not in the drinking driver’s car. Manning *et al.* [30] take a slightly less rigid view, including also costs paid by governments and others for medical and pension costs of heavy drinkers. But basically it can be concluded that the rigid conceptual boundaries used in studies in this tradition make them peripheral to the task of estimating and understanding harms to others from a drinker’s drinking.

The mainline cost of alcohol studies have tended to divide their estimates according to the societal response institutions from which their primary data mainly derive. The recent Swedish study categorized its main direct costs into: health care, social services, and crime [31, p. 71]. The most recent Australian study by Collins and Lapsley [28] subtracts welfare and adds costs of work defaults (and road accidents, elsewhere covered under crime). A Finnish study adds “material damage” from traffic accidents, fire and crimes to the basic list of health, welfare and crime [32, p. 136].

So far we have been dealing with what economists call the “direct costs” of alcohol consumption— costs of the major societal response institutions which, at least in a welfare-state society, are primarily borne by the society. To the extent this is true, those around the drinker—family, friends, victims, bystanders—are not much out of pocket for the actual costs which are measured—though they do contribute to paying them as taxpayers. However, in the economists’ conceptualization, there are two categories of cost beyond what we have considered which do have a more direct impact on those around the drinker.

One of these is the indirect costs or the productivity costs – the loss to the economy from a drinker’s early death or other incapacity to work. Typically these costs, primarily from early deaths, are the largest or second-largest component of cost of alcohol calculations [31, p. 95]. The other category is intangible costs, in which a cost is assigned to pain and suffering, and more generally a diminished quality of life. The inclusion of both these categories in cost of alcohol estimates is controversial [31, pp. 14–15]; the standard solution at present is to include indirect costs but not intangible costs.

If we consider these categories in terms of the harm of drinking to others, both have substantial implications, particularly for members of the drinker’s immediate family or household. An early death or disability due to alcohol results in the reduction of net resources (wealth) used in paying for funeral expenses, health costs or legal proceedings – in essence direct costs. But the death or disability also removes from the family budget the earnings of the drinker, often substantially reducing the family’s future earnings (*i.e.*, indirect costs). Finally, early death and disability are also likely to cause distress in the family and impact one’s quality of life (intangible costs). In these senses, what is measured as “lost productivity” is also an indication of harm to others. Likewise, there are likely to be substantial intangible costs of living with a heavy drinker. One widely-used measure of intangible health costs is Quality-of-Life Years (QALYs), with a full-quality year of life scored at 1.0 and death at 0, and mental and physical health disablements scored in between. A substudy of the Swedish cost of alcohol study estimated the loss in terms of QALYs for those who reported someone close to them or living in their household having a drinking problem, finding a significant decrement among those sharing a household with someone with a drinking problem. Assigning the standard costing used in Swedish economic studies to the QALYs, the estimated intangible cost to those sharing a house was 10.9 billion Swedish crowns, compared with total net direct and indirect costs to the society (from health, crime, lost productivity and early mortality) of 20.3 billion [31, pp. 69,71].

**Figure 2.**
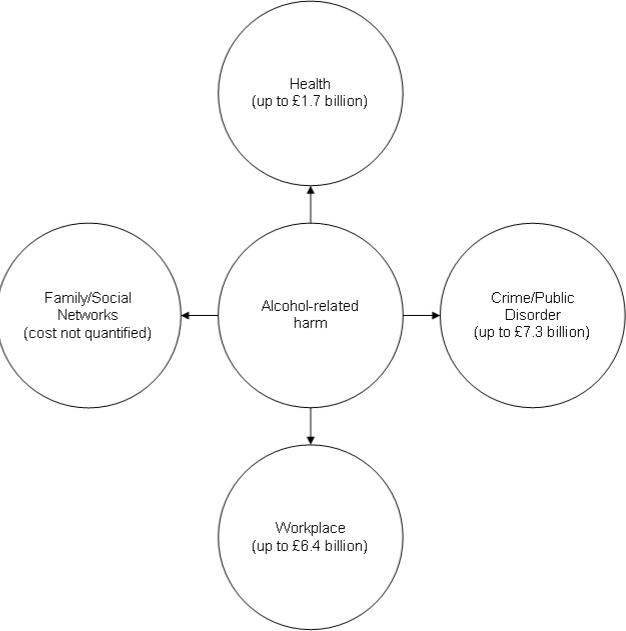
Domains of social costs of alcohol (according to [33]).

These results underline a point made in the cost of alcohol study for England and Wales [33]. The study divided the main social costs into four categories: health; crime/public disorder; workplace; and family/social network (Figure 2). Although it was able to quantify costs only for the first three categories, it insisted on noting the fourth as an important category.

There are several sources of this lacuna in the cost of alcohol studies. Often (as in the English study) even costs in the welfare system are omitted. This is partly because the proportion of welfare caseloads which could be attributed to someone’s drinking is not well measured anywhere, and not measured at all in most places. Also reflected are economists’ rules on what counts and what does not count in a cost study, which exclude “transfer costs” (costs which do not add or subtract any wealth to the society), including most welfare payments.

Also involved in the neglect of family/social network costs is the old tradition in economics of treating the household as a consumer unit and as “the primary decision making unit in our society” [29]. As Johansson *et al.* [31, p. 17] note, “arguments have been made that consumption decisions are made within the family, which would imply that no external costs could arise within a family”—although these authors reject this argument, noting that choices about drinking reflect among other things “the bargaining position of family members”. Lastly, there is the problem of assigning costs to “intangible” dimensions such as quality of life, and an understandable hesitation of researchers to add together the tangible costs of running a societal response system and such intangible costs.

The conclusion, in the context of cost of alcohol studies, must thus be that “social harms, such as problems in family life and personal friendships and relationships, have not been well measured” [31, p. 11]. Beyond this, it may be that a social-cost approach may not be the best way to quantify such harms.

Mainstream cost of alcohol studies often provide some subdivisions of the costs. For instance, Collins and Lapsley [28, pp. 68,69] estimate that, of the $10.8 billion tangible social costs of alcohol in Australia in 2004/5, 25% are paid by governments—$1.3 billion by the federal government and $1.4 billion by state governments. But no attempt is usually made to split costs to others from costs to the drinker. In terms of direct costs (excluding productivity losses from early deaths), Collins and Lapsley estimate that, of the social costs of alcohol, 9.2% are for work absenteeism, 35.6% for health care, 29.4% for road traffic accidents, and 25.7% for crime (recalculated from [28, pp. 60,64]). If we take as costs not borne by the drinker all of the crime costs and one-third of the other direct costs in these estimates, we might guess that about one-half of the costs are “externalities”, costs which are not borne by the drinker. This rough estimation does not specify who is bearing these costs.

## 5. Approaches to Studying Alcohol’s Harm to Others

An important step in studying alcohol’s harm to others is to ascertain what data can be found or developed concerning the adverse effects of someone’s drinking on specific others. The adverse effects on the other can be in terms of mental or physical health, of safety or security, or tangible or intangible costs. The effects can be as described by the other, and noted by a bystander or interested party, or as recorded in agency records. The data can be drawn from multiple sources, which do not necessarily share common definitions or frames of reference. Thus the harms that are measured are not necessarily mutually exclusive—though an interesting question, of course, is what can be said about overlap and potential doublecounting?

Harm to an individual can occur in many forms, and can be measured in various metrics. Likewise, alcohol’s involvement in the harm can be established or estimated in several ways. For instance:

1. The fact of an occurrence, for instance, a death or a traffic crash, can be viewed as an indicator of harm for the person affected. Thus a count of deaths, or of years of life lost (YLLs) short of a full life, is one kind of metric of harm to the individual.
2. Particularly in population survey data, the respondent’s report of an adverse occurrence—for example, something being broken or damaged that mattered to the respondent—is often used as an indication of harm to the respondent.
3. The frequency and intensity of such harms can also be indicated by the respondent, for instance by asking how often it happened, and whether the adverse effect was a lot or a little.
4. Another measure used of the degree of harm to the respondent can be in terms of the respondent’s report on items indicating a degree of wellbeing or of health disability (e.g., impact on health related quality of life, HRQoL), which can then be compared with the reports of others similarly situated.
5. For harms where it is possible, a monetary measure of the amount of harm can be calculated. This can be measured in various frames of reference, depending on the harm: e.g., in terms of the wages which could have been earned in time which was lost, in terms of the loss of monetary value of something broken or damaged, in terms of the conventional monetary value assigned by econometric studies to an increment of health disability.

Costs of the effect of someone’s drinking on another which are not paid by the affected person can also be estimated—costs which are paid collectively, usually by a government, such as hospitalization or policing costs.

The definition of causation which will be commonly used in such studies is an epidemiological one [4]: would the adverse event have happened in the absence of the drinking? The drinking is thus neither necessary nor sufficient for the event to have occurred, and other factors will often have also played a causal role. But, viewed from a policy perspective, the definition answers the crucial question: would removing the drinking have prevented the adverse event?

1. In data drawn from population surveys, alcohol’s causal involvement in the harm is commonly measured directly by the attribution of the person affected. Thus a question like “How many times in the last 12 months were you physically hurt by them because of their drinking?” has built into the question an attribution (in a “yes” response) of the occurrence to the other’s drinking.
2. For some harms, the alcohol attribution is commonly made by the personnel of the health or social response system—for instance, a child protection worker coding that the parent’s drinking is involved in a child endangerment case. Sometimes the attribution is built into the system’s categorization of the case—for instance, a Foetal Alcohol Syndrome diagnosis, or a “drunk and disorderly” arrest.
3. For harms measured by the health system without any routine coding of alcohol involvement, meta-analyses of special studies have often relied on to measure an “alcohol attributable fraction” (AAF). AAFs are usually concerned with the drinking of the person with the illness, but it is possible to develop analogous fractions concerning the drinking of another, for instance, in estimating the proportion of child traffic deaths where another’s drinking played a causal role.
4. In studying the effects of others’ drinking on personal wellbeing and health disability, the alcohol attribution can be imputed from differences in wellbeing or disability scores between those similarly situated who are affected by heavy drinkers and those who are not.

All of these metrics of harm and means of assessing causality have a substantial base in the research literature. But all are the subject of continuing debate and discussion, as scientists continue to endeavour to improve concepts and measurement.

## 6. Getting the Right Focus in a Binocular View

Like the social cost studies, a study of alcohol’s harm to others will normally use data collected in two main frames. One frame is that of surveys of the general population; the other is that of agency records, such as police and ambulance files, hospitalisation and emergency department records, alcohol treatment agency casefiles, and mortality records.

Each frame has its own characteristics, advantages and drawbacks. The advantage of the population survey is that in principle it is inclusive of the whole range of experience in the population, including lesser problems which would never come to official attention. The drawbacks include that really serious adverse effects are not very common, and it would take a very large sample of the population to cover them adequately. The picture of adverse effects drawn from a population survey is thus going to be dominated by the milder end of the continuum of severity of effects. A second drawback is that it is increasingly impossible to get an adequate representation of the whole population in a sample survey. Marginalised people are often missing from a sample based on households, and increasingly young adults are missing from survey samples defined by having a landline telephone. Also, response rates have fallen as populations have found themselves bombarded not only with research surveys but also with telemarketing masquerading as a survey.

Agency records have the advantage that they are likely to give a good picture of the more serious end of the continuum of severity. In contrast to population studies, one drawback is that less serious cases tend not to come to an agency’s attention. A second drawback is that the data is usually collected as part of the agency staff’s routine work activities; in many cases, the staff is involved in life-and-death situations, and accurate data recording will often come lower on the priority list. Details like the involvement of someone’s drinking in the situation may not be routinely recorded. Alternatively, if there is a required tick-box concerning this, the box will be filled out, but its validity may be questionable. What is recorded may also be shaped by how the staff member wants to present their daily work to their supervisors and others who may use the records.

Using data from both these two main frames gives the opportunity for what might be called a binocular view of alcohol’s harm to others. In studies of those whose drinking causes problems, one finds what has been called “two worlds of alcohol problems” [34]. Looked at through the general-population frame, alcohol problems are fairly dispersed, and, for most of those with problems, the problems are relatively mild. On the other hand, in populations showing up in institutional frames— hospitals, the police, social welfare agencies—the problems are often more serious and cumulative. Particularly in alcohol treatment agencies, those with alcohol problems are also often highly marginalized—much less likely to be in the workforce, to be stably housed, to be in a family relationship [35]. The “two worlds” division may be less marked among those affected by others’ drinking than among drinkers themselves, but needs to be kept in mind as an issue.

The challenge for future work in the field, then, is not only to develop better measurement of problems as they are manifested both through agency windows and through the general population window, but also to develop study designs which chart and limn in the connections. When and under what circumstances does the risk increase that a minor annoyance on the street will turn into a serious event? What can be learned about prodromal signs that the wellbeing of family members is seriously threatened by another member’s drinking?

## 7. Conclusion

From court records, coroner’s studies and the casefiles of emergency response agencies, we know that drinking can have very serious adverse effects on those around the drinker. From limited sets of items on population surveys, we know that others’ drinking sometimes annoys or discomforts a very large fraction of the population. We need to know more about the scope and magnitude of these problems at both ends of the spectrum of severity, and we need to know what lies between these extremes: the circumstances and prevalence of adverse effects which are not cataclysmic, but which also cannot be shrugged off. We need to know more about the natural history of relationships where the drinking of at least one person has an adverse effect on others. When do relationships persist despite heavy drinking and when do they lapse? What are the longer-term effects of one person’s heavy drinking on the other, in a particular type of relationship? To what extent and under what circumstances are drinkers responsive to efforts at social control from others in a social relationship? These kinds of questions suggest how inherently social and interactional is the study of the harmful effects of drinking on others, and of efforts by the other or by third parties to prevent or reduce the harm. Drinking is usually a social activity, and drinkers interact with and affect those in their social environment.

The knowledge is of practical value in terms of designing effective resources to counsel and assist those adversely affected by others’ drinking. Generally, provisions for those seeking such help are presently an add-on to services for those with alcohol problems. A fuller and more concrete understanding of the harms to others from drinking is also important in designing prevention programs and policy initiatives to reduce the harms from drinking. It also has a potentially important role in policy debates about public health approaches to alcohol problems. Some will argue that what the drinker does to his or her own body and mind is private rather than public business. But even those who take a very restrictive view of the responsibilities of the state will commonly agree that the state should intervene to prevent harm from one person’s behaviour to another.
